# Impact of the *rpoS *genotype for acid resistance patterns of pathogenic and probiotic *Escherichia coli*

**DOI:** 10.1186/1471-2180-7-21

**Published:** 2007-03-26

**Authors:** Sina M Coldewey, Maike Hartmann, Dorothea S Schmidt, Uta Engelking, Sya N Ukena, Florian Gunzer

**Affiliations:** 1 Institute for Medical Microbiology and Hospital Epidemiology, Hannover Medical School, Carl-Neuberg-Strasse 1, 30625 Hannover, Germany; 2 Department of Anesthesiology, Hannover Medical School, Carl-Neuberg-Strasse 1, 30625 Hannover, Germany; 3 Helmholtz Centre for Infection Research, Department of Mucosal Immunity, Inhoffenstrasse 7, 38124 Braunschweig, Germany; 4 Technische Universität Dresden, Faculty of Medicine Carl Gustav Carus, Institute for Medical Microbiology and Hygiene, Medical Theoretical Centre, Fiedlerstraße 42, 01307 Dresden, Germany

## Abstract

**Background:**

Enterohemorrhagic *E. coli *(EHEC), a subgroup of Shiga toxin (Stx) producing *E. coli *(STEC), may cause severe enteritis and hemolytic uremic syndrome (HUS) and is transmitted orally via contaminated foods or from person to person. The infectious dose is known to be very low, which requires most of the bacteria to survive the gastric acid barrier. Acid resistance therefore is an important mechanism of EHEC virulence. It should also be a relevant characteristic of *E. coli *strains used for therapeutic purposes such as the probiotic *E. coli *Nissle 1917 (EcN). In *E. coli *and related enteric bacteria it has been extensively demonstrated, that the alternative sigma factor σ^S^, encoded by the *rpoS *gene, acts as a master regulator mediating resistance to various environmental stress factors.

**Methods:**

Using *rpoS *deletion mutants of a highly virulent EHEC O26:H11 patient isolate and the sequenced prototype EHEC EDL933 (ATCC 700927) of serotype O157:H7 we investigated the impact of a functional *rpoS *gene for orchestrating a satisfactory response to acid stress in these strains. We then functionally characterized *rpoS *of probiotic EcN and five r*poS *genes selected from STEC isolates pre-investigated for acid resistance.

**Results:**

First, we found out that ATCC isolate 700927 of EHEC EDL933 has a point mutation in *rpoS*, not present in the published sequence, leading to a premature stop codon. Moreover, to our surprise, one STEC strain as well as EcN was acid sensitive in our test environment, although their cloned *rpoS *genes could effectively complement acid sensitivity of an *rpoS *deletion mutant.

**Conclusion:**

The attenuation of sequenced EHEC EDL933 might be of importance for anyone planning to do either *in vitro *or *in vivo *studies with this prototype strain. Furthermore our data supports recently published observations, that individual *E. coli *isolates are able to significantly modulate their acid resistance phenotype independent of their *rpoS *genotype.

## Background

Enterohemorrhagic *Escherichia coli *(EHEC), a subgroup of Shiga toxin (Stx) producing *E. coli *(STEC), are enteric pathogens frequently causing severe illness in humans. EHEC infection may lead to non-bloody and bloody diarrhea and most dangerously, the extraintestinal complication hemolytic uremic syndrome (HUS) [1,2]. In order to cause gastrointestinal disease, bacteria must pass through the acidic gastric barrier. When taking into account the apparent low infectious dose of enterohemorrhagic *E. coli*, which may be as little as 100 viable organisms [3], it becomes obvious that acid resistance is an important virulence trait of EHEC. Investigation of the complex mechanisms conferring acid tolerance onto *E. coli *has revealed many new insights in the last years. Currently, four different acid stress protection systems are being discussed. At least three of these are controlled by σ^S^, an alternative sigma subunit of RNA polymerase encoded by the *rpoS *gene [4]. σ^S^, which is seen as a master regulator of general stress response, confers promoter specificity to the core RNA polymerase and is activated under a variety of stress conditions, as well as during stationary phase [5-7]. The glutamate-dependent acid resistance (GDAR) system is believed to provide best protection for bacterial cells below pH 3 [8]. Bhagwat et al. have recently reported, that in natural populations of pathogenic *E. coli *mutations in *gadE *exist which, in addition to mutant alleles of *rpoS*, may contribute to varying acid resistance phenotypes of EHEC [9]. *gadE *encodes the GadE protein, a regulatory molecule of the GDAR system [10].

In 1994 Small et al. already reported that the growth pH is important for expression of acid resistance in *E. coli *[11]. Waterman et al. who have investigated a large set of STEC for acid resistance [12], identified defective *rpoS *genes as cause for loss of acid resistance in individual *E. coli *isolates. Recently it was demonstrated by King et al., that modulation of genome usage enables regulatory diversity which contributes to strain variation in *E. coli *[13]. A similar observation was reported by Bhagwat et al., who investigated stress tolerance of EHEC and could observe a functional heterogeneity of RpoS [14].

Our study was initially focused on analyzing the role of σ^S ^in acid stress behavior of two different EHEC isolates used as model organisms in our laboratory: the sequenced prototype O157:H7 EHEC EDL933 (ATCC 700927) [15] and a very well characterized EHEC O26:H11 isolate from a HUS patient [16,17]. We expected these results to improve the interpretation of data obtained from *in vitro *and *in vivo *virulence experiments investigating pathogen-host interactions. Using homologous recombination and suicide vector technique we constructed unmarked isogenic *rpoS *deletion mutants of both strains. Resistance of wild type organisms and their mutants towards acidic conditions at pH 2.5 and 1.5 were tested as described by Lange et al. [18] with slight modifications. While the EHEC O26:H11 patient isolate was highly dependent on a functional *rpoS *gene for an adequate response to acid stress, surprisingly, deletion of *rpoS *in EHEC EDL933 had no measurable effect when compared to its wild type strain. Further investigation revealed a point mutation in the EHEC EDL933 *rpoS *gene which leads to a TAA stop codon being responsible for this phenotype.

We then evaluated another 39 isolates of human and porcine origin and functionally characterized a subgroup of five selected r*poS *genes. Surprisingly, σ^S ^activity of STEC ED-68 [19,20] appeared to be dependent on a yet unknown regulatory mechanism that modulated its activity. In the following, we could also observe a similar phenomenon with the well characterized probiotic *E. coli *Nissle 1917 [21-23].

## Results

### Construction and confirmation of unmarked isogenic *rpoS *deletion mutants

After PCR screening of potential mutants one *rpoS *negative isolate of each EHEC wild type strain, EDL933_a _and 126814 (Table 1), was subjected to further testing by Southern blotting. They both showed DNA fragments of the expected size, after restriction digest of their genomic DNA with either *Xmn*I or *Stu*I [see Additional file 1] thereby indicating the correct insertion of the *rpoS *deletion mutation into the genome of the two EHEC strains. The mutants were termed *E. coli *MHH933-5 and MHH126-5 respectively. Additionally, the mutation was confirmed by DNA sequencing of a PCR product generated with primers RpoS 3 and RpoS 4. Antibiotic resistance patterns of the mutants did not differ from their parental strains. Biochemical reaction profiles of all strains, as determined with API 20 E strips, were quite similar [see Additional file 2]. EHEC EDL933_a _and its mutant, which both could not ferment sorbitol, were identified as 89.6 % *E. coli *according to their API profile index "5144172". Sorbitol positive EHEC 126814 and mutant strain *E. coli *MHH126-5 generated the profile index "5144562" which was 99.8 % specific for *E. coli*. Additionally, all *E. coli *O157:H7 isolates were able to ferment rhamnose, while the O26:H11 strains were not. After 1 h treatment with 30 mM hydrogen peroxide no viable bacteria could be recovered from cultures of *E. coli *MHH933-5, MHH126-5 and EHEC EDL933_a_, while EHEC 126814 and EDL933_b _(Table 1) still produced more than 60 % colony forming units (CFU) compared to the blank value (data not shown).

### Acid resistance assays

**Figure 1:**
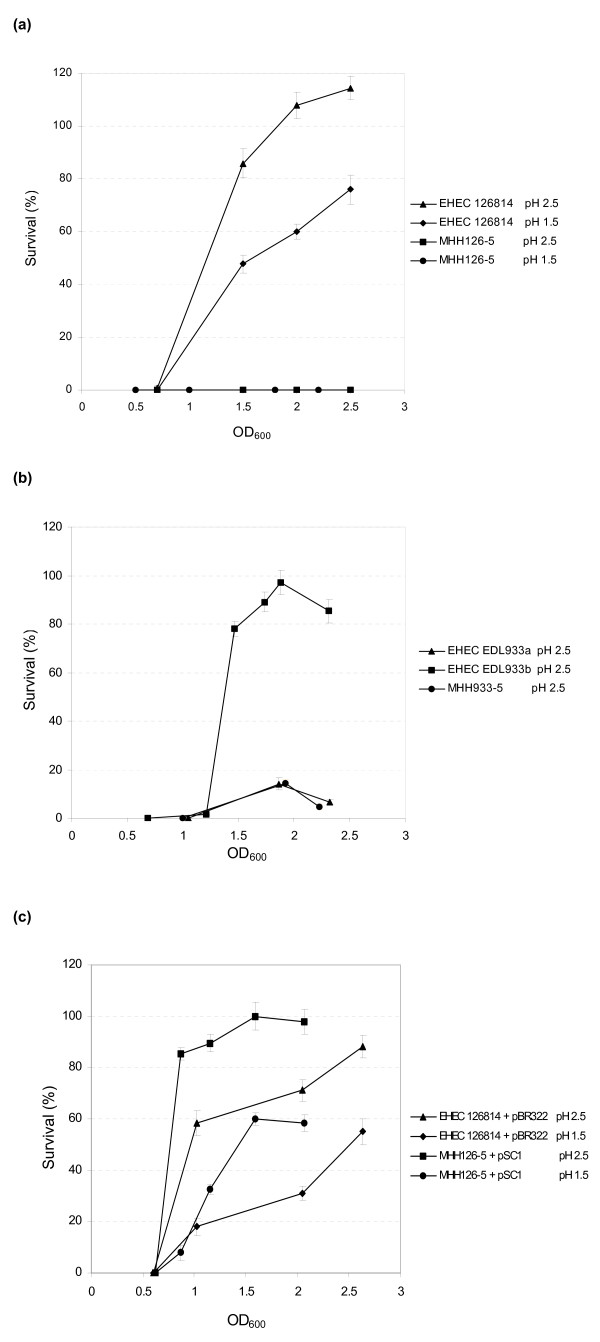
**A: Acid resistance of EHEC 126814 and *E. coli *MHH126-5**. Inducible acid resistance of EHEC wild type strain 126814 and its isogenic *rpoS *deletion mutant *E. coli *MHH126-5 was investigated after 2 h incubation in LB media at pH 2.5 or 1.5. The wild type strain showed a high level of acid resistance, which was induced from OD_600 _0.7 of the preparatory culture. It reached up to 115 % survival at OD_600 _2.5 of the starter culture, indicating that EHEC 126814 was able to grow under these conditions. In LB media with pH 1.5 up to 75 % of the inoculum survived. *E. coli *MHH126-5 was completely sensitive to acid treatment regardless of pH and OD_600 _of the preparatory culture. Percentage survival figures in relation to OD_600 _of one typical experiment are depicted. The means and standard deviations were calculated from three independent dilution series made at each individual measuring point. **B: Acid resistance of EHEC EDL933_a_, EDL933_b _and *E. coli *MHH933-5**. EHEC EDL933_b _was very acid resistant in all experiments. However, between EHEC wild type strain EDL933_a _and its mutant *E. coli *MHH933-5 no differences could be observed. Both isolates showed weak resistance under acidic growth conditions and showed a similar behavior in all other experiments. In contrast to EHEC 126814, acid resistance of the O157 isolates was induced at OD_600 _1.2 of the starter culture. One typical experiment has been shown as a representation of all independent tests carried out. The means and standard deviations were calculated from three independent dilution series prepared at each individual measuring point. **C: Acid resistance of *E. coli *MHH126-5 complemented with pSC1**. This figure depicts the inducible acid resistance in relation to OD_600 _of one typical experiment. Acid resistance was assayed at pH 2.5 and 1.5. By complementation of *rpoS *deletion mutant *E. coli *MHH126-5 with pSC1, containing its own *rpoS *gene cloned into plasmid pBR322, a phenotype could be restored that was even more resistant to acid stress than wild type strain EHEC 126814. OD_600 _of acid resistance induction was identical to values obtained with the wild type strain, shown in figure 1A. In order to compare growth conditions in LB media containing ampicillin, positive control EHEC 126814 had been transformed with plasmid pBR322. Compared to figure 1A, the antibiotic and/or pBR322 negatively influenced acid resistance of this strain. The percentage survival at pH 2.5 was below 90 %. One typical experiment is shown representative of independent tests. The means and standard deviations were calculated from three independent dilution series made at each individual measuring point.

The strains became acid resistant from OD_600 _0.7 for EHEC 126814 and from OD_600_ 1.2 for EHEC EDL933_a_, respectively. EHEC 126814 very effectively responded to acid stress (Fig. 1A) and showed resistance rates up to 115 % at pH 2.5, indicating bacterial growth at this low pH, and 75 % at pH 1.5. In contrast, EHEC EDL933_a _was only moderately acid resistant at pH 2.5 as was its isogenic Δ*rpoS *mutant (Fig. 1B). The survival of these strains was about 17 % at most, which appeared to be independent of the *rpoS *genotype. The behavior of a second clone of EHEC EDL933_a_, obtained from ATCC, was identical (data not shown). EHEC EDL933_b _however, was nearly as acid resistant as EHEC 126814 (Fig. 1A and 1B). Furthermore, *E. coli *MHH126-5 was completely unable to survive acidic growth conditions at pH 2.5 and pH 1.5 (Fig. 1A). By complementation of *E. coli *MHH126-5 with pSC1 bearing its own *rpoS *gene, a wild type like phenotype could be restored. At pH 2.5 it was even more resistant than the parental organism. At pH 1.5 its survival was still about 60 % (Fig. 1C). When complemented with pSC2, harboring rpoS*, the *rpoS *gene from EHEC EDL933_a_, *E. coli *MHH126-5 was completely sensitive to acidic growth conditions further on (data not shown). However, when *E. coli *MHH126-5 was transformed with plasmid pMH33, containing an rpoS* allele cured by site directed mutagenesis from the TAA stop codon at position 723, its acid resistance increased to more than 100 % (data not shown). Thus, we could unequivocally prove that the point mutation G721T in rpoS* was solely responsible for the acid sensitive phenotype of EHEC EDL933_a_.

### Homology comparison of *rpoS *genes

Homology of all sequenced *rpoS *genes was compared with the software package BioEdit [see Additional file 3]. In the following all nucleotide positions are enumerated as described in materials and methods. We again sequenced the *rpoS *gene of EHEC EDL933_b _and used it as reference for all comparisons since the respective nucleotide data from the genome sequence NC_002655 had two sequencing errors, G57C and G61C. The main *rpoS *promoter *rpoS*p is located within the *nlpD *gene at position -568 to -566. The 35 and the -10 region are positioned at -601 to -596 and -578 to -573 respectively. Both, the highly acid resistant EHEC strain 126814 as well as the STEC isolate E-D53 had the point mutation G-570A, located in between the 10 region and *rpoS*p. Furthermore, these strains had point mutation A-521G leading to an amino acid exchange from threonine to alanine in NlpD. EHEC 126814 and 288597, STEC E-D53 and E-D68 and EcN also showed mutations T-306C in the *nlpD *gene, as well as A543C in *rpoS*. With the exception of EHEC 288597, these strains were mutated at position T387C as well. EHEC 126814 and the two STEC strains E-D53 and E-D68 also carried mutation A819G. Furthermore, EHEC 126814 and STEC E-D53 had the additional mutation G-465A in *nlpD*. STEC E-D68 showed four more point mutations: C-472T, which leads to an amino acid exchange from threonine to isoleucine in NlpD, C-293T, G-183T and A-162G. EcN carried the nucleotide exchange C-463T resulting in isoleucine instead of threonine in NlpD. Furthermore, the *rpoS *gene of EcN had point mutations T171C, C272T, T365G, T470C, T581C, and C995T. Except for the three amino acid exchanges in NlpD described above, all other point mutations observed in either *rpoS *or *nlpD *of all *E. coli *strains investigated were silent. In contrast, EHEC 86-24 had an 8 bp duplicate sequence (GAAGAGGA) in *rpoS *beginning at position 131 which caused a shift in the open reading frame and a stop codon 219 bp later.

### Functional analysis of further *rpoS *genes

**Figure 2:**
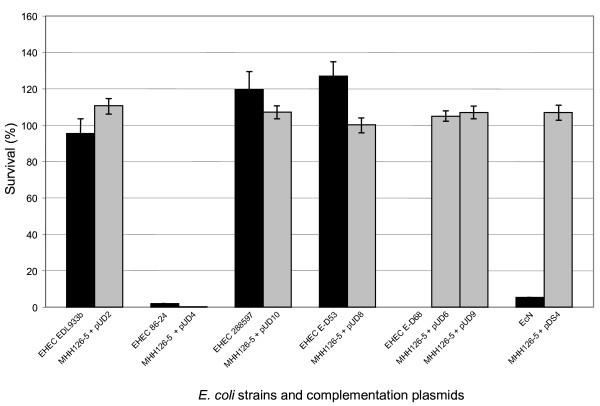
**Functional analysis of further *rpoS *genes by complementation of *E. coli *MHH126-5**. This figure shows resistance data of the *rpoS *deletion mutant *E. coli *MHH126-5 complemented with each of the plasmids pUD2 (pBR322 + *rpoS*_EDL933b_), pUD4 (pBR322 + *rpoS*_86-24_), pUD10 (pBR322 + *rpoS*_288597_), pUD8 (pBR322 + *rpoS*_E-D53_), pUD6 (pBR322 + *rpoS*_E-D68_), pUD9 (pBR322 + *rpoS*_E-D68_) or pDS4 (pBR322 + *rpoS*_EcN_) in comparison to the corresponding wild type EHEC strains EDL933_b_, 86-24 and 288597, STEC isolates E-D53 and E-D68 as well as EcN. All wild type strains are indicated by black bars, the complemented mutants by grey ones. Plasmids pUD2 and pUD4, pUD10 and pUD8 conferred an acid resistance phenotype to the mutant, which was comparable to the corresponding parental strain. Interestingly, upon complementation with pUD6 and pUD9, bearing the *rpoS *gene of STEC E-D68, *E. coli *MHH126-5 became strongly pH resistant. This was in sharp contrast to the behavior of the STEC E-D68 wild type strain. A similar phenomenon was observed when the acid resistance test strain *E. coli *MHH126-5 was complemented with pDS4 containing *rpoS *of EcN. The means and standard deviations were calculated from three independent dilution series in this exemplary experiment.

All experiments are illustrated in figure 2. Plasmids pUD2, pUD8 and pUD10 conferred a highly acid resistant phenotype on to *E. coli *MHH126-5, comparable to the respective wild type organisms EHEC EDL933_b_, EHEC 288597 and STEC E-D53. As expected, complementation with pUD4 did not mediate acid resistance to *E. coli *MHH126-5. This behavior was comparable to the parental strain EHEC 86-24. However, when the test strain *E. coli *MHH126-5 was transformed with pUD6, surprisingly it became strongly acid resistant. This was in sharp contrast to the manner of the corresponding wild type strain STEC E-D68, which only showed an acid resistance ≤ 0.1 %. This response becomes comprehensible considering the unaffected open reading frame in the *rpoS *sequence of pUD6. To confirm this effect we constructed pUD9, a second independent plasmid containing *rpoS *from STEC E-D68. With this plasmid the acid resistance of complemented *E. coli *MHH126-5 was identical to pUD6. Interestingly, we observed a similar phenomenon when we investigated acid tolerance of probiotic EcN. While survival of the wild type organism after pH 2.5 treatment was only around 5 %, test strain *E. coli *MHH126-5 became fully acid resistant, when complemented with plasmid pDS4 containing *rpoS *of EcN (Fig. 2).

## Discussion

Gastric acid is a natural barrier that all bacteria entering the lower intestine have to pass. With pH values ranging between 1.5 and 2.5, the stomach is one of the most inhospitable areas in the human body. A correlation between an infectious dose of enterobacteriaceae and their capacity to withstand acidic conditions is well known. With respect to the very low infectious dose of EHEC, of about 100 to 1000 organisms [3], it becomes obvious, that acid resistance is a key factor for virulence of these bacteria. *E. coli *have developed elegant regulatory systems, that enable their survival under such conditions [4,24]. The alternative sigma factor σ^S ^is instrumental in the regulation of acid protection mechanisms in this species of enterobacteriacae [4].

In this study we have investigated the role of the *rpoS *genotype on the acid stress response of a set of Shiga toxin producing *E. coli *as well as the widely used probiotic *E. coli *strain Nissle 1917 [21-23]. We first constructed unmarked isogenic *rpoS *deletion mutants of the highly virulent EHEC patient isolate 126814 [16,17] and of the completely sequenced prototype EHEC EDL933 [15], labeled EHEC EDL933_a _in this study. The patient isolate was highly dependent on a functional *rpoS *gene for an adequate response to acid stress. By complementation of its *rpoS *deletion mutant *E. coli *MHH126-5 with the *rpoS *gene from EHEC 126814 a wild type like phenotype could be restored. Surprisingly, EHEC EDL933_a _and its mutant did not exhibit major differences regarding their acid tolerance, but both appeared to be σ^S ^defective. Indeed, when we sequenced rpoS* from EHEC EDL933_a _we could identify a point mutation that caused a premature stop codon which was in conflict with the published database sequence [15]. In order to rule out that this mutation was an artifact generated in our laboratory, we purchased a second isolate of sequenced EHEC EDL933. It carried the identical point mutation, which may have occurred during passage prior to storage of the isolate at the strain collection. Allelic variations in the *rpoS *gene are not uncommon since it is localized in a highly mutable region of the *E. coli *genome [25,26]. In a large study Waterman et al. identified EHEC strains that were defective in their response to low pH [12]. They attributed this phenotype to a non functional σ^S ^as a consequence of *rpoS *mutations. Phenotypical characterization of EHEC EDL933_b_, a further clone of EDL933, and complementation experiments clearly showed that the stop codon in rpoS* causally determined the stress phenotype of ATCC strain 700927. When considering the significant impact of σ^S ^in the regulatory network of *E. coli*, which controls up to 10 % of the *E. coli *genes directly or indirectly [27], this observation is of importance for those working in the field of EHEC and planning to do both, *in vitro *or *in vivo *studies with this particular sequenced isolate.

Further investigation of six *rpoS *genes from Shiga toxin producing *E. coli *and a probiotic *E. coli *strain revealed that σ^S ^activity is not always dependent on the *rpoS *genotype. Despite having a functional *rpoS *gene, as shown by complementation experiments in a Δ*rpoS *background, STEC E-D68 as well as EcN behaved σ^S ^defective regarding their acid resistance. While STEC E-D68 was completely sensitive to acid stress, probiotic EcN exhibited about 5 % survival in the same test environment. With respect to EcN, this observation adds to an actual study of Bhagwat et al. where the authors observed functional heterogeneity of σ^S ^in food-borne and clinical EHEC isolates [14]. However, in STEC E-D68 as well as in EcN, mutations in the gene encoding the GDAR system regulator GadE [10] have to be ruled out. Such mutations have been reported by Bhagwat et al. as another reason for attenuated acid resistance in *E. coli *wild type isolates [9]. With regard to the varying σ^S ^activities in individual *E. coli *strains observed in our study, it should be mentioned that all isolates analyzed here carried glutamate at codon 33 (GAG) resulting in σ^S ^(33E). This seems to account for a higher variability in *rpoS *related phenotypes as recently described by Atlung et al. [25].

Price et al. have shown recently, that EHEC make use of their different acid resistance systems depending on the type of acid stress they are exposed to [28,29]. In either case σ^S ^was important for a sufficient acid stress response of EHEC EDL933 (ATCC 43895) *in vitro *as well as *in vivo *[29]. It should be investigated though, whether this impact of σ^S ^on the *in vivo *acid resistance of EHEC also allows assumptions about the virulence of a particular strain. In our laboratory environment for instance wild type EHEC 86-24 was highly virulent in an oral infection model with gnotobiotic piglets [16], although it has a non functional *rpoS *gene and is only weakly acid resistant as shown above. Krogfelt et al. have published an accomplished experiment which clearly demonstrates that *rpoS *gene function may be a disadvantage for *E. coli *colonizing the intestine [30]. The authors therefore conclude that the benefit of a functional *rpoS *regulon for *E. coli *depends on the actual growth phase of a particular strain. The apparent down regulation of σ^S ^activity in STEC E-D68 and EcN in our study seems to be another way of adapting the *rpoS *regulon to specific growth conditions. We have performed further experiments to investigate the impact of *rpoS *in EcN on regulation of potential host probiotic marker genes which we have recently identified [31]. Our long-term objective is to establish to what extent *E. coli *is able to modulate pathogenic but also beneficial properties using its *rpoS *regulatory network.

## Conclusion

The results of our study clearly confirm the central role of σ^S ^as a key regulator for acid resistance in STEC and EcN. When interpreting *in vitro *or *in vivo *data generated with EHEC EDL933_a _it is important to realize, that this prototype EHEC has an attenuated σ^S ^phenotype. We could also show that *rpoS *gene function is modified in singular *E. coli *isolates by regulatory mechanisms that lead to an altered σ^S ^activity, as exemplified by STEC strain E-D68 and probiotic EcN. This is in line with the observations regarding functional heterogeneity of RpoS in stress tolerance of K-12 *E. coli *strains and EHEC isolates, King et al. and Bhagwat et al. have reported [13,14], and expands them to a commensal *E. coli *with beneficial traits.

## Methods

### Bacterial strains and media

All bacterial strains investigated in detail in this study are listed in table 1. Acid stress response assays were performed with EHEC 126814, a highly virulent patient isolate [16,17] and the prototype O157:H7 EHEC EDL933. Two independent clones of EHEC EDL933 were used, the sequenced strain ATCC 700927 [15], referred to as EHEC EDL933_a _and the original isolate ATCC 43895/BCCM LMG 15068 [32], termed EHEC EDL933_b_. Acid resistance was also evaluated using 39 human and porcine EHEC and STEC strains (data not shown) as well as the probiotic *E. coli *Nissle 1917 [21-23]. Unmarked isogenic *rpo*S negative mutants were produced from EHEC EDL933_a _and from EHEC 126814. *E. coli *SM 10 λ*pir *is a λ lysogen of *E. coli *SM10 and contains the trans acting factors needed to replicate and mobilize all suicide plasmids [33] used in this study. *E. coli *DH5α (Invitrogen, Karlsruhe, Germany) was used as host strain for all other plasmids. Bacteria were grown in LB broth (Invitrogen) and on MacConkey agar (Oxoid, Wesel, Germany), MH agar (Becton Dickinson, Heidelberg, Germany) or LB agar plates (Invitrogen) at 37°C or on LB sucrose plates (10 % sucrose w/v, no NaCl) at room temperature. Ampicillin (Ratiopharm, Ulm, Germany) was added at a concentration of 200 μg/ml, where necessary. Concentrated HCl was used to adjust LB broth for acid resistance testing at pH 2.5 or 1.5. Chemicals were obtained from Sigma, Deisenhofen or Merck, Darmstadt, Germany.

### Primers and sequencing

Table 2 lists all oligonucleotide primers used in this study. These were synthesized by MWG-Biotech (Ebersberg, Germany), who also completed the custom sequencing of PCR products and plasmids. Sequences of all *nlpD-rpoS *genes investigated in this study were generated by primer walking with two independent PCR products for which corresponding accession numbers are given in table 3. Nucleotide data was analyzed with the DNASTAR software package (Lasergene, DNASTAR, Madison, WI, USA), the BioEdit Sequence Alignment Editor 7.0.5.3. [34] and Clone Manager 6 (Scientific & Educational Software, Cary, NC, USA) and then submitted to GenBank. Nucleotide positions within *rpoS*, *nlpD *or the flanking regions are referenced to the open reading frame of *rpoS*. Positions 5' upstream of the *rpoS *start codon are indicated with a negative sign, positions 3' downstream of the stop codon with a positive one.

### Plasmids and DNA preparation

**Figure 3:**
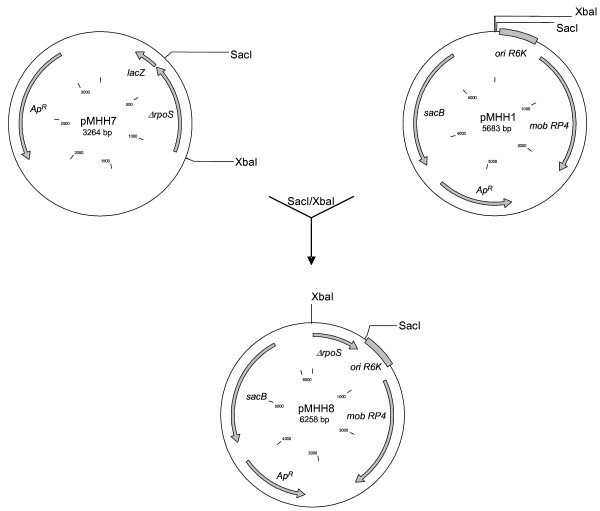
**Construction of suicide vector pMHH8**. The *rpoS *gene from EHEC 86-24 was amplified by PCR with primers RpoS 3/RpoS 4 and cloned into pUC19 after restriction digest with enzymes *Xba*I and *Sac*I (pMHH6). A 390 bp sequence was deleted from the insert through restriction digest with enzymes *Dra*III and *Bsa*AI leading to plasmid pMHH7. The mutated *rpoS *gene was then cloned into suicide vector pMHH1 after restriction digest with *Xba*I and *Sac*I. The resulting plasmid was termed pMHH8. It was used in the construction of all *rpoS *deletion mutants described in this study.

**Figure 4:**
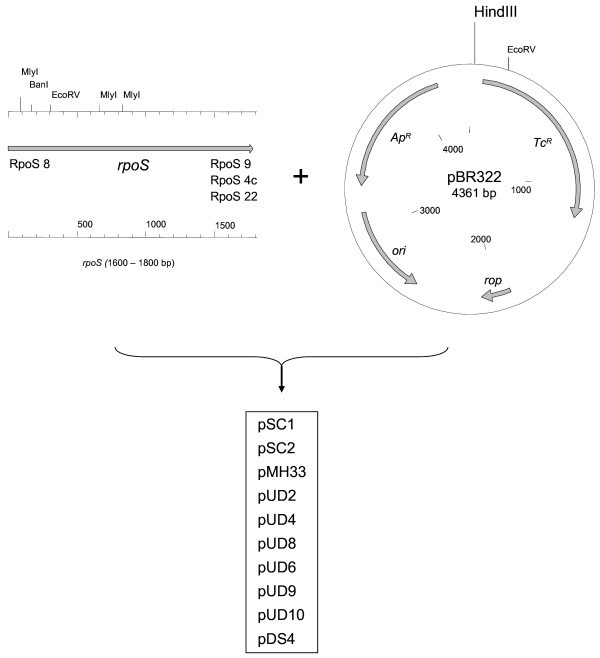
**Construction of complementation plasmids**. Complementation plasmids pSC1, pSC2, pUD2, pUD4, pUD8, pUD6, pUD9, pUD10 and pDS4 were constructed using low copy vector pBR322 as backbone. Each of them harbors one complete *rpoS *gene and the *rpoS*p promoter [39] from EHEC/STEC strains 126814 (pSC1), EDL933_a _(pSC2), EDL933_b _(pUD2), 86-24 (pUD4), E-D53 (pUD8) and 288597 (pUD10), amplified by PCR with primers RpoS 8/RpoS 9 containing 5' *Hind*III restriction sites. RpoS 8 and RpoS 4c were taken to synthesize the respective DNA fragment from STEC E-D68 in order to generate the two independent plasmids pUD6 and pUD9. pDS4 contained the cloned *rpoS *gene from EcN, amplified with primers RpoS 8 and RpoS 22. pMH33 was made from pSC2 by PCR mediated site specific mutagenesis, employing the mutation *rpoS*_T721G _to resolve the TAA stop codon in rpoS*. RpoS negative mutant strain *E. coli *MHH126-5 was transformed with each of the complementation vectors by electroporation.

All plasmids used in this study are listed in table 3. Allelic exchange experiments were conducted with the suicide vector pMHH8 derived from plasmid pGP704 [33]. A map of suicide vectors pMHH1, pMHH7 and pMHH8 is shown in figure 3. All other cloning was performed in pUC19 [35] or pBR322 [36]. Construction of all complementation plasmids is depicted in figure 4. Plasmid DNA was isolated using the Qiagen Plasmid Midi Kit (Qiagen, Hilden, Germany). Chromosomal DNA was purified with Qiagen-tip 100 columns and the Genomic DNA Buffer Set from Qiagen.

### Suicide vector pMHH8

For construction of suicide vector pMHH8 the *rpoS *gene from EHEC 86-24 [37] was amplified by PCR using primers RpoS 3/RpoS 4 and cloned into pUC19. Upon restriction digest with enzymes *Dra*III and *Bsa*I (New England Biolabs, Schwalbach, Germany) a 390 bp deletion in *rpoS *was created. The mutated gene was then subcloned into pMHH1, a derivative of suicide vector pGP704. The resulting plasmid was called pMHH8 (Fig. 3). It contained a positive selection system based on the *sacB *gene from *Bacillus subtilis *[38] to facilitate screening for potential mutants. Electroporation was used to transform *E. coli *SM 10 λ*pir *with pMHH8.

### Construction of complementation plasmids

For complementation experiments, an *nlpD-rpoS *DNA amplicon, containing the *rpoS *main promoter *rpoS*p [39] was generated by PCR using primers with 5' *Hind*III restriction sites (Table 2). RpoS 8 and RpoS 9 were taken to amplify *nlpD*-*rpoS *from EHEC strains 126814 (pSC1), EDL933_a _(pSC2), EDL933_b _(pUD2), 86-24 (pUD4), 288597 (pUD10) and from porcine STEC strain E-D53 (pUD8). RpoS 8 and RpoS 4c were needed to synthesize the respective DNA fragment from porcine STEC E-D68 (pUD6 and pUD9). Amplification of *nlpD*-*rpoS *from EcN (pDS4) was performed with primers RpoS 8 and RpoS 22. Following endonuclease digest, the amplicon was cloned into the low copy plasmid pBR322 linearized with *Hind*III (New England Biolabs). Plasmid pMH33 was produced from pSC2 by the PCR mediated nucleotide exchange T721G with primers RpoS 17a/RpoS 17b using the QuikChange Site Directed Mutagenesis Kit from Stratagene (Amsterdam, The Netherlands) according to manufacturer's instructions. *rpoS *deletion mutant *E. coli *MHH126-5 was then transformed with each of the complementation vectors by electroporation.

### Construction of *rpoS *deletion mutants from EHEC EDL933_a _and EHEC 126814

Allelic exchange using suicide vector pMHH8 was performed in a two-step procedure as previously described [40]. A positive selection system based on the *sacB *gene from *Bacillus subtilis *[38] was used to facilitate screening for potential mutants.

### Genotypical and phenotypical confirmation of mutant strains

The *rpo*S deletion mutation was confirmed by the sequencing of PCR products obtained with primers RpoS 3 and RpoS 4, as well as with Southern hybridizations of chromosomal DNA from EHEC EDL933_a _and EHEC 126814 wild type and mutant strains digested with either *Stu*I or *Xmn*I (New England Biolabs) [see Additional file 1]. Hybridization was performed under high stringency conditions with digoxigenin labelled probes using the Dig Labeling and Detection Kit from Roche Diagnostics (Mannheim, Germany). Specific binding was detected by autoradiography and chemoluminescence with CSPD (Applied Biosystems, Darmstadt, Germany) as substrate. API 20 E strips (bioMérieux Deutschland, Nürtingen, Germany) were used to analyze biochemical profiles of mutants and their parental strains. Antibiotic resistances of all isolates were determined with the MERLIN Microdilution Detection System (MICRONAUT-SB, MERLIN Diagnostika, Bornheim-Hersel, Germany). Resistance of EHEC EDL933_a_, EDL933_b_, 126814 and of the mutants *E. coli *MHH933-5 and MHH126-5 to 30 mM hydrogen peroxide was tested as described by Lange et al. [18]. Viability of bacteria was evaluated after 5, 10, 20, 30 and 60 minutes of H_2_O_2 _treatment.

### Acid resistance assays

Inducible acid resistance of EHEC EDL933_a_, EHEC EDL933_b_, EHEC 126814, the mutants *E. coli *MHH933-5 and MHH126-5 as well as of *E. coli *MHH126-5 complemented with pSC1, pSC2 or pMH33, was assessed by inoculating 100 ml LB broth pH 7.0 1:1000 with an overnight culture from each bacterial strain. Bacteria were sampled at defined time points, as depicted in figure 1A to 1C, and subjected to incubation at pH 2.5 or 1.5 for two hours. Colony forming units (CFU) of untreated and acid treated cultures were determined. Acid resistance screening of further EHEC and STEC isolates, of EcN and of *E. coli *MHH126-5 complemented with pUD2, pUD4, pUD6, pUD8, pUD9, pUD10 or pDS4 was performed in a similar manner but as a one step test at pH 2.5, with cultures grown to an OD_600 _of 1.7 (Fig. 2). Acid resistance was calculated as percentage CFU recovered after acid exposure compared to untreated cultures. Resistance data has been based on three independent tests.

### Complementation experiments with mutant *E. coli *MHH 126-5

*E. coli *strain MHH126-5 was transformed with each of the complementation plasmids by electroporation. Inducible resistance at pH 2.5 or 1.5 was assayed with *E. coli *MHH126-5 complemented with pSC1 (Fig. 1C), using the same experimental procedure as described above, except for adding 200 μg/ml ampicillin to all media. Functional activity of pSC2, pMH33 and all other cloned *rpoS *genes (Fig. 2) was evaluated with the one step test described above for acid resistance screening. EHEC 126814 transformed with pBR322 was used as reference strain for these experiments.

## Abbreviations

ATCC, American Type Culture Collection; BCCM, Belgian Co-ordinated Collections of Microorganisms; CFU, colony forming units; EHEC, enterohemorrhagic *E. coli*; HUS, hemolytic uremic syndrome; EcN, *E. coli *Nissle 1917

## Competing interests

The author(s) declare that they have no competing interests.

## Authors' contributions

SMC carried out all of the experiments described except for functional characterization of additional *rpoS *genes from STEC 288597, E-D53 and E-D68 and EcN, was involved in the interpretation of data, designed figures and tables and wrote the manuscript. MH constructed plasmid pMH33, used it for complementation experiments and participated in the analysis of sequenced *rpoS *genes. DSS sequenced and functionally characterized *rpoS *of EcN. UE screened a collection of STEC strains for acid resistance and has sequenced and functionally characterized *rpoS *genes from STEC strains 288597, E-D53 and E-D68. SNU has performed preparatory investigations to this study, which were instrumental in designing the subsequent experiments. FG is primary investigator, who conceived the study, helped to interpret the data and critically revised and finished the manuscript. All authors have read and approved the final manuscript.

## Supplementary Material

Additional file 1 Genotypical confirmation of *rpoS *mutants by Southern blotting. In this figure genomic DNA is shown digested with either *Xmn*I (A) or *Stu*I (B). DNA from wild type strains EHEC EDL933_a _and 126814 is separated in lanes 1 and 3, DNA from the corresponding mutants *E. coli *MHH933-5 and *E. coli *MHH126-5 is running in lanes 2 and 4 respectively. The probes were hybridizing with fragments of a calculated size of 1042 bp (wild type) and 652 bp (mutants) after restriction digest with *Xmn*I (A) and 841 bp (wild type) and 451 bp (mutant), when *Stu*I (B) was used. No additional bands appeared. kb = DNA Molecular Weight Marker III, DIG-labelled (Roche Molecular Biochemicals, Mannheim, Germany). Click here for file

Additional file 2 Biochemical reaction profiles in the API 20 E test. This table displays the biochemical reactions of EHEC EDL933, EHEC 126814 and their isogenic *rpoS *negative mutants as determined with API 20 E strips. Click here for file

Additional file 3 Multiple alignment of *rpoS *alleles. A multiple nucleotide sequence alignment of all eight *rpoS *alleles functionally characterized and described in detail in this study is provided in BioEdit format. The file can be viewed with the sequence alignment editor BioEdit [34]. The program is available free of charge at [42]. Click here for file

## Figures and Tables

**Table 1:** Bacterial strains investigated in this study.

**Laboratory code**	***stx *genotype**	**Serotype**	**Reference**
EDL933_a _(ATCC 700927)	*stx*_1_, *stx*_2_	O157:H7	[15]
EDL933_b _(ATCC 43895/LMG 15068)	*stx*_1_, *stx*_2_	O157:H7	[32]
126814/97	*stx*_2_	O26:H11	[16,17]
86-24	*stx*_2_	O157:H7	[37]
E-D53	*stx*_2e_	O101: H^-^	[19,20]
E-D68	*stx*_2e_	O101: H^-^	[19,20]
288597/03	*stx*_2_	O157:H^-^	This study
Nissle 1917	∅	O6:H1	[21-23]

All bacterial strains investigated in detail in this study are listed with their laboratory codes, *stx *genotype, serotype and references, if applicable.

**Table 2:** Oligonucleotide primers used in this study.

**Target gene**	**Name**	**Position**	**5' Sequence 3'**	⇔
*rpoS*	RpoS 3	4 – 24	GCT CTA GAA GTC AGA ATA CGC TGA AAG TT *Xba*I	s
*rpoS*	RpoS 4	989 – 969	CCG AGC TCC TCG CGG AAC AGC GCT TCG AT *Sac*I	as
*rpoS*	RpoS 4c	1129 – 1109	CCC AAG CTT TTA CTC GCG GAA CAG CGC TTC *Hind*III	as
*rpoS*	RpoS 5	572 – 589	ATG AAC CAA GTG GG AAG	s
*rpoS*	RpoS 6	871 – 854	ACA TCT TCC AGT GTT GCC	as
*nlpD*	RpoS 8	(- 657) – (- 636)	CCC AAG CTT CGA CGC AGC AGA GCA AGG AGT T *Hind*III	s
*rpoS*	RpoS 9	1141 – 1119	CCC AAG CTT CAG CTG GTG CGC AAG ATG ATG AA *Hind*III	as
*rpoS*	RpoS 17a	706 – 736	GAC ATC CTG GCC GAT **G**AA AAA GAG AAC GGT C	s
*rpoS*	RpoS 17b	736 – 706	GAC CGT TCT CTT TTT **C**AT CGG CCA GGA TGT C	as
*rpoS*	RpoS 22	(+ 171) – (+ 151)	CCC AAG CTT GCC AAA TGT GAC GCT GAC GCG *Hind*III	as

Primers used in this study are listed with their 5' – 3' sequences. All nucleotide positions refer to the start codon of their target gene. Positions of RpoS primers within *rpoS*, *nlpD *or the flanking regions are referenced to the open reading frame of *rpoS*. Positions 5' upstream of the start codon are indicated with a negative sign, positions 3' downstream of the stop codon with a positive one. s = sense, as = antisense

**Table 3:** Plasmids used in this study.

**Plasmid**	**Description**	**Host**
pUC19	• High copy plasmid [41]; accession # L09137 • Amp^R^	• *E. coli *DH5α
pGP704	• Suicide vector [33] • Backbone from pBR322, *ori *R6K instead of *ori *E1 from pBR322, *mob *region from pRP4 • Replicates in *E. coli *SM10λ*pir *but not in EHEC strains used because *ori *R6K needs π-Protein coded by the *pir *gene for replication • Amp^R^	• *E. coli *SM10λ*pir*
pMHH1	• Derivative of pGP704 • Has a positive selection system based on the *sacB *gene from *Bacillus subtilis *[38]	• *E. coli *SM10λ*pir*
pMHH6*	• Derivative of pUC19 • Harbors a 990 bp *rpoS *fragment from EHEC 86-24 amplified with primers RpoS 3/RpoS 4 and cloned via *Xba*I und *Sac*I restriction sites	• *E. coli *DH5α
pMHH7*	• Derivative of pMHH6 • Restriction digest with *Dra*III und *Bsa*AI leads to a 390 bp deletion in *rpo*S	• *E. coli *DH5α
pMHH8*	• Derivative of pMHH1 • Contains the mutagnized 600 bp *rpoS *fragment from pMHH7 cloned via *Xba*I and *Sac*I restriction sites	• *E. coli *SM10λ*pir*
pBR322	• Low copy plasmid [36]; accession # J01749 • Amp^R^, Tet^R^	• *E. coli *DH5α • EHEC 126814
pSC1*	• Derivative of pBR322 • Harbors *nlpD-rpoS *from EHEC 126814 accession # DQ272417 amplified by PCR using primers RpoS 8/RpoS 9 and cloned via *Hind*III restriction sites	• *E. coli *DH5α • *E. coli *MHH126-5
pSC2*	• Derivative of pBR322 • Harbors *nlpD-rpoS *from EHEC EDL933_a _(rpoS*) accession # DQ272419 amplified by PCR with primers RpoS 8/RpoS 9 and cloned via *Hind*III restriction sites	• *E. coli *DH5α • *E. coli *MHH126-5
pMH33*	• Made from pSC2 by PCR mutagenesis using Primers RpoS 17a/RpoS 17b • Has point mutation *rpoS*_T721G _which resolves the TAA stop codon at position 723 in rpoSEDL933a MathType@MTEF@5@5@+=feaafiart1ev1aaatCvAUfKttLearuWrP9MDH5MBPbIqV92AaeXatLxBI9gBaebbnrfifHhDYfgasaacH8akY=wiFfYdH8Gipec8Eeeu0xXdbba9frFj0=OqFfea0dXdd9vqai=hGuQ8kuc9pgc9s8qqaq=dirpe0xb9q8qiLsFr0=vr0=vr0dc8meaabaqaciaacaGaaeqabaqabeGadaaakeaaieGacqWFYbGCcqWFWbaCcqWFVbWBcqWFtbWudaWgaaWcbaacbaGae4xrauKae4hraqKae4htaWKae4xoaKJae43mamJae43mamZaaSbaaWqaaiabdggaHbqabaaaleqaaaaa@39CB@	• *E. coli *MHH126-5
pUD2*	• Derivative of pBR322 • Harbors *nlpD-rpoS *from EHEC EDL933_b _accession # DQ272421 amplified by PCR with primers RpoS 8/RpoS 9 and cloned via *Hind*III restriction sites	• *E. coli *MHH126-5
pUD4*	• Derivative of pBR322 • Harbors *nlpD-rpoS *from EHEC 86-24 accession # DQ272418 amplified by PCR with primers RpoS 8/RpoS 9 and cloned via *Hind*III restriction sites	• *E. coli *MHH126-5
pUD8*	• Derivative of pBR322 • Harbors *nlpD-rpoS *from STEC E-D53 accession # DQ272423 amplified by PCR with primers RpoS 8/RpoS 9 and cloned via *Hind*III restriction sites	• *E. coli *MHH126-5
pUD6*	• Derivative of pBR322 • Harbors *nlpD-rpoS *from STEC E-D68 accession # DQ272424 amplified by PCR with primers RpoS 8/RpoS 4c and cloned via *Hind*III restriction sites	• *E. coli *MHH126-5
pUD9*	• Derivative of pBR322 • Harbors *nlpD-rpoS *from STEC E-D68 accession # DQ272424 amplified by PCR with primers RpoS 8/RpoS 4c and cloned via *Hind*III restriction sites	• *E. coli *MHH126-5
pUD10*	• Derivative of pBR322 • Harbors *nlpD-rpoS *from EHEC 288597 accession # DQ272422 amplified by PCR with primers RpoS 8/RpoS 9 and cloned via *Hind*III restriction sites	• *E. coli *MHH126-5
pDS4*	• Derivative of pBR322 • Harbors *nlpD-rpoS *from EcN amplified by PCR with primers RpoS 8/RpoS 22 and cloned via *Hind*III restriction sites	• *E. coli *MHH126-5

All plasmids used in this study are listed with their names, descriptions and host strains. Sequenced *rpoS *genes were submitted to GenBank. Their accession numbers are given in the table. * = plasmid designed in this study
