# Supplementary material for: Impact of the rpoS genotype for acid resistance patterns of pathogenic and probiotic Escherichia coli
Source: BMC Microbiol. 2007 Mar 26;7:21. doi: 10.1186/1471-2180-7-21 (PMC1852560; doi:10.1186/1471-2180-7-21)
Supplement: Additional file 2 — Biochemical reaction profiles in the API 20 E test. This table displays the biochemical reactions of EHEC EDL933, EHEC 126814 and their isogenic rpoS negative mutants as determined with API 20 E strips. [file 1471-2180-7-21-S2.doc]

**Biochemical reaction profiles in the API 20 E test.**

| **Identification code** | **Reaction** | **EHEC EDL933_a_**  **EHEC EDL933_b_**  ***E. coli* MHH933-5** | **EHEC 126814**  ***E. coli* MHH126-5** |
| --- | --- | --- | --- |
| ONPG | o-nitrophenyl-β-galactosidase | + | + |
| ADH | Argininedehydrolase | - | - |
| LDC | Lysinedecarboxylase | + | + |
| ODC | Ornithinedecarboxylase | + | + |
| CIT | Citrate | - | - |
| H_2_S | H_2_S | - | - |
| URE | Urease | - | - |
| TDA | Tryptophanedeaminase | - | - |
| IND | Indole | + | + |
| VP | Voges-Proskauer reaction | - | - |
| GEL | Gelatinase | - | - |
| GLU | Glucose fermentation | + | + |
| MAN | Mannitol fermentation | + | + |
| INO | Inositol fermentation | - | - |
| SOR | Sorbitol fermentation | **-** | **+** |
| RHA | Rhamnose fermentation | **+** | **-** |
| SAC | Saccharose fermentation | + | + |
| MEL | Melibiose fermentation | + | + |
| AMY | Amygdalin fermentation | - | - |
| ARA | Arabinose fermentation | + | + |
| API profile index |  | 5144172 | 5144562 |
| Species identification |  | *E. coli* 1 = 89,6 % | *E. coli* 1 = 99,8 % |

Biochemical reactions of O157:H7 EHEC EDL933, O26:H11 EHEC 126814 and their mutants as determined with API 20 E strips. Positive reactions are indicated with a “+” sign, negative ones with a “-“. The isolates from the two serogroups only differ in their ability to ferment sorbitol and rhamnose.
