# Supplementary material for: Impact of the rpoS genotype for acid resistance patterns of pathogenic and probiotic Escherichia coli
Source: BMC Microbiol. 2007 Mar 26;7:21. doi: 10.1186/1471-2180-7-21 (PMC1852560; doi:10.1186/1471-2180-7-21)
Supplement: Supplementary file 2 [file 1471-2180-7-21-S1.doc]

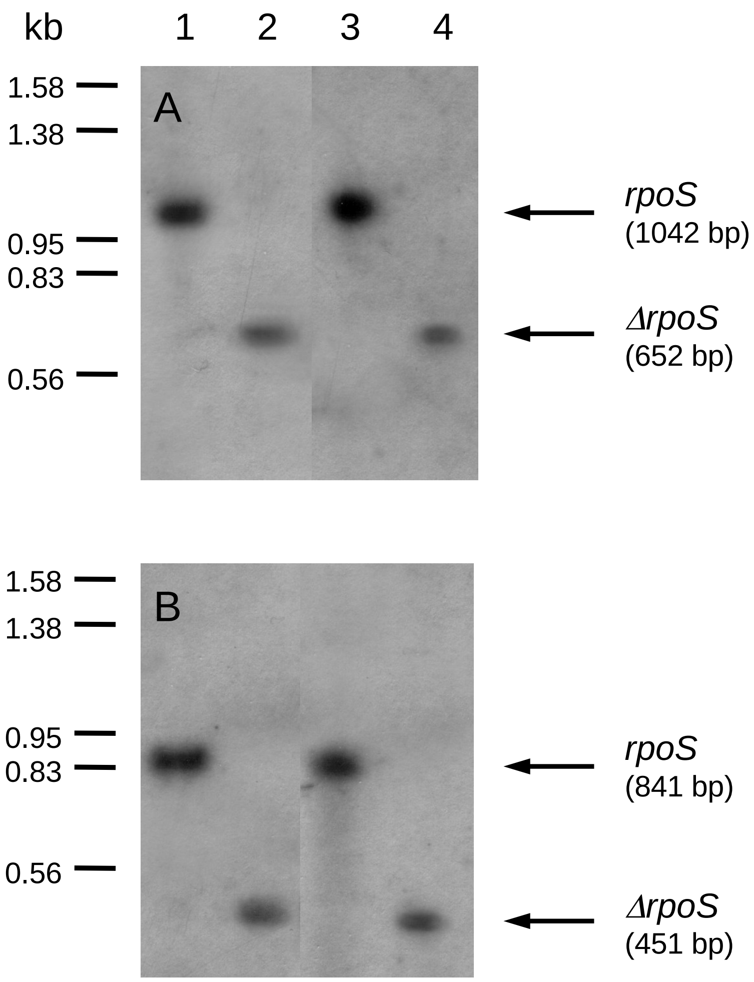
kb
1
2
3
4
A
1.58
1.38
0.95
0.83
0.56
rpoS
(1042 bp)
ΔrpoS
(652 bp)
B
1.58
1.38
0.95
0.83
0.56
rpoS
(841 bp)
ΔrpoS
(451 bp)
